# CRISPR/Cas-Based Gene Editing Strategies for DOCK8 Immunodeficiency Syndrome

**DOI:** 10.3389/fgeed.2022.793010

**Published:** 2022-03-17

**Authors:** Sujan Ravendran, Sabina Sánchez Hernández, Saskia König, Rasmus O. Bak

**Affiliations:** Department of Biomedicine, Aarhus University, Aarhus, Denmark

**Keywords:** gene editing (CRISPR-Cas9), CRISPR/Cas 9, hematopoietic stem cell, DOCK8 immunodeficiency syndrome, DOCK8 deficiency, DOCK8, gene therapy, Primary immunodeficiency

## Abstract

Defects in the DOCK8 gene causes combined immunodeficiency termed DOCK8 immunodeficiency syndrome (DIDS). DIDS previously belonged to the disease category of autosomal recessive hyper IgE syndrome (AR-HIES) but is now classified as a combined immunodeficiency (CID). This genetic disorder induces early onset of susceptibility to severe recurrent viral and bacterial infections, atopic diseases and malignancy resulting in high morbidity and mortality. This pathological state arises from impairment of actin polymerization and cytoskeletal rearrangement, which induces improper immune cell migration-, survival-, and effector functions. Owing to the severity of the disease, early allogenic hematopoietic stem cell transplantation is recommended even though it is associated with risk of unintended adverse effects, the need for compatible donors, and high expenses. So far, no alternative therapies have been developed, but the monogenic recessive nature of the disease suggests that gene therapy may be applied. The advent of the CRISPR/Cas gene editing system heralds a new era of possibilities in precision gene therapy, and positive results from clinical trials have already suggested that the tool may provide definitive cures for several genetic disorders. Here, we discuss the potential application of different CRISPR/Cas-mediated genetic therapies to correct the DOCK8 gene. Our findings encourage the pursuit of CRISPR/Cas-based gene editing approaches, which may constitute more precise, affordable, and low-risk definitive treatment options for DOCK8 deficiency.

## Introduction

Primary immunodeficiencies (PIDs) include more than 400 rare congenital monogenic disorders characterized by impairment of immunity, susceptibility to infectious diseases, autoimmunity, autoinflammatory diseases, allergy and/or malignancy (Tangye et al., 2020). In recent years, there has been an increase in the recognition and diagnosis of previously undefined genetically caused abnormalities in the immune system (Tangye et al., 2020). This has been made possible through the completion of the Human Genome project in the early 2000s, improved definition of clinical phenotypes, and advancement of cost-effective and time-efficient sequencing through implementation of next generation DNA sequencing technologies (Meyts et al., 2016; Bousfiha et al., 2020; Tangye et al., 2020; Gates et al., 2021).

Among these disorders is DOCK8 immunodeficiency syndrome (DIDS) also known as DOCK8 deficiency. Until recently DOCK8 deficiency was termed DOCK8-related Hyper Immunoglobulin E (IgE) Syndrome (HIES), as it is characterized by elevated IgE levels, eosinophilia, and recurrent infections. The majority of HIES is caused by either autosomal dominant inheritance (AD-HIES) of mutations in the signal transducer and activator of transcriptase 3 (STAT3) gene (Grimbacher et al., 1999), or autosomal recessive inheritance (AR-HIES) primarily of mutations in the guanine-nucleotide exchange factor dedicator of cytokinesis 8 (DOCK8) gene (Su et al., 2011). However, an increased insight into the functionality of DOCK8 illuminates its impact on both the T- and B-cell compartment of the immune system, which has promoted the reclassification as a DOCK8-related combined immunodeficiency (CID). DOCK8 deficiency is a severe disorder with early onset of morbidity and high mortality rates exceeding those associated with STAT3 HIES (Aydin et al., 2015; Tsilifis et al., 2021). Since the majority of the clinical manifestations of DOCK8 deficiency pertain to the immune system, hematopoietic stem cell transplantation (HSCT) in early childhood is encouraged. However, this is challenged by the need for a HLA-matched donor and associated with adverse events such as immune rejection and graft-versus-host disease (Copelan, 2006; Aydin et al., 2019).

The ideal treatment of DOCK8 deficiency would be correcting the disease-causing mutation in the patient’s own cells, thereby restoring the DOCK8 functionality. This would circumvent the obstacle of identifying HLA compatible donors for allogeneic transplantation and eliminate the associated risks. During the past two decades, genetic therapies have shown promising results for an expanding numbers of genetic disorders (Booth et al., 2019; Porteus, 2019). Meanwhile, precise genome editing tools were developed and applied in a range of pre-clinical and even a few clinical gene therapy studies. In particular, the discovery of the CRISPR/Cas system as a highly versatile genome editing platform accelerated the development of genome editing methods (Bak et al., 2018a). This ultimately led to the 2020 Nobel Prize in Chemistry awarded to Emmanuelle Charpentier and Jennifer Doudna for ‘the development of a method for genome editing’. CRISPR/Cas offers unprecedented simplicity in facilitating genome editing, and has proven highly precise and efficient (Jensen et al., 2019; Porteus, 2019). Since the first injections of CRISPR gene edited cells into patients in 2016 (Cyranoski, 2016), there have been published reports on only a few clinical trials. These trials have marked important milestones by providing evidence on safety, whereas recent clinical trials on sickle cell disease, β-thalassemia, and transthyretin amyloidosis have been the first to demonstrate therapeutic and potentially curative potential (Frangoul et al., 2021; Gillmore et al., 2021).

In preclinical studies, CRISPR/Cas gene editing has shown tremendous potential in a wide range of diseases, but has so far not been applied to DOCK8. Here, we will elaborate on why gene editing is within the realm of possibility for treating DOCK8 deficiency. First, we briefly present our current understanding of the genetic, molecular, and cellular mechanisms involved in DOCK8 deficiency. Second, we portray the common disease manifestations and discuss current diagnostic and treatment approaches. Third, after describing recent advancements in the field of genome editing and discussing advantages and disadvantages of the different precise gene editing platforms, we define suitable CRISPR/Cas strategies for treating, which may constitute a definitive cure for DOCK8 deficiency. Finally, we give a concise summary of hurdles and challenges for using gene editing in the clinical setting.

## The Genetics of DOCK8 Deficiency

The large DOCK8 gene is located on the short arm of chromosome 9, includes 48 exons, spans over 250 kilobases, and encodes a protein of approximately 190 kDa. Bi-allelic loss-of-function mutations in the DOCK8 gene is associated with DOCK8 deficiency (Zhang et al., 2010; Database resources of the, 2018). DOCK8 deficiency is estimated to affect less than one person per million, but the exact prevalence is unknown (Biggs et al., 2017). The disease was not recognized until 2009, and only about 200 cases have been described world-wide so far, which have been identified predominantly in populations with consanguineous marriage (Zhang et al., 2009; Biggs et al., 2017).

**FIGURE 1:**
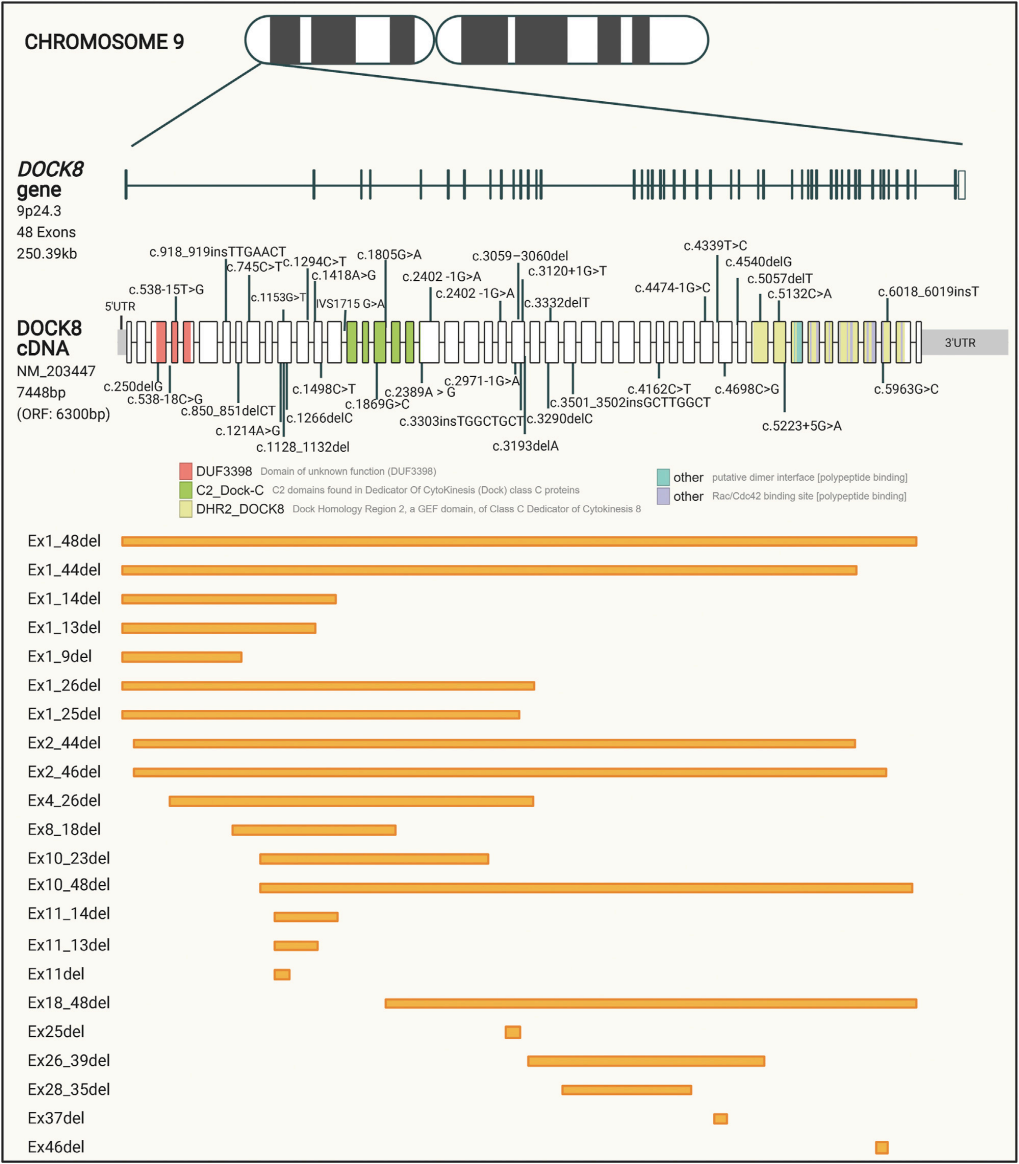
Schematic representation of reported patient mutations in the DOCK8 gene. The DOCK8 gene, composed of 48 exons, is located on the short arm of chromosome nine and spans over 250 kb. The distribution of mutations associated with DOCK8 deficiency collected from the ClinVar database is represented along the DOCK8 cDNA. Boxes represent the 48 exons of the gene and different colors indicate major domain-encoding regions. Out of a total of 1,139 DOCK8 variants reported to date, 60 have been found in patients where DOCK8 deficiency has been diagnosed. Orange boxes represent deletions of one or more exons.

To get a collected overview on the different patient mutations, we performed a comprehensive data collection of **60** disease-causing DOCK8 variants described in the literature and registered in the ClinVar database. These variants are represented in Figure 1 and listed in Supplementary Table S1. Even though no specific mutation hotspot regions were identified, the majority of disease-causing mutations in DOCK8 were deletions which cover 61.5% of the variants and range from a few base pairs to deletions spanning several hundred base pairs. The high propensity for deletions has been hypothesized to be partly caused by the occurrence of repetitive genomic sequences leading to abnormal recombinations in this region (Engelhardt et al., 2009). The pathogenic variants in DOCK8 are predominantly loss of function, thus abolishing the expression of DOCK8, but occurrences of DOCK8 duplication has been shown to associate with neurodevelopmental conditions (Jing et al., 2014).

As with a few other primary immunodeficiencies, there have been reported cases of somatic reversion leading to partial re-expression of DOCK8 protein in some cell linages. These occurrences of “natural gene-therapy” may reflect the location of DOCK8 within a recombination hotspot, promoting either a somatic repair of a point-mutation, recombination-mediated gene conversion, or recombination-mediated intragenic single crossover (Pillay et al., 2021). Jing et al. observed some clinical improvement in seventeen patients with somatic reversion, with significant improvement in overall survival and age-stratified morbidity. However, these improvements were insufficient for disease elimination presumed to be due to inadequate DOCK8 re-establishment, particularly within the T cells (Pillay et al., 2021). In contrast, Pillay et al. identified three patients with biallelic compound heterozygous DOCK8 germline variants, who displayed significant DOCK8 expression in their lymphocyte subsets ranging from 10% of all B cells to 75% of all CD8^+^ T cells, while myeloid cells did not express DOCK8. DNA sequencing analyses revealed that one pathogenic allele had been genetically repaired, which was hypothesized to have occurred in either a single common lymphoid progenitor cell or a single hematopoietic stem cell. In all three patients, the somatic reversion improved survival, differentiation, and function of lymphocytes and provided great clinical improvement to the patients (Namekata et al., 2014). Such single progenitor/stem cell reversions signify that only modest DOCK8 correction frequencies by gene therapy in autologous hematopoietic stem cells or lymphoid progenitor cells could provide significant clinical benefit to the patients.

## The Role of DOCK8

Until recently, the molecular mechanism of DOCK8 and its influence in cell homeostasis was unknown and unexplored. However, recent discoveries have shed light on these, and we present here these recent discoveries with a focus on the immunoregulatory influence of DOCK8.

### Molecular Homeostasis of DOCK8

DOCK8 belongs to the subfamily of DOCK proteins, which are atypical guanine nucleotide exchange factors (GEFs), which to date consists of 11 proteins numerically named from DOCK1 to 11 (Côté and Vuori, 2007). DOCK proteins activate small G proteins (guanine nucleotide-binding proteins), which are GTPases involved in signal transduction. G proteins bind GTP in their on-state but hydrolyse GTP to GDP and then transition into an off-state. Re-activation requires dissociation of GDP and binding of GTP—an exchange that is facilitated by DOCK proteins. DOCK proteins consist of two conserved protein domains known as Dock Homology Region 1 and 2, (DHR1 and -2) (Côté et al., 2005). The DHR1 domain is located upstream of the DHR2 domain and mediates the binding to phosphoinositide (PI), which leads to localized GEF activity near the cell membrane (Sakurai et al., 2021; Côté and Vuori, 2002). The DHR2 domain interacts with the nucleotide-free form of Rho GTPases such as RhoA, Rac, or Cdc42, depending on the DOCK protein. This interaction induces the catalytic activation of the GTPases mediated by GDP-GTP exchange (Harada et al., 2012).

**FIGURE 2:**
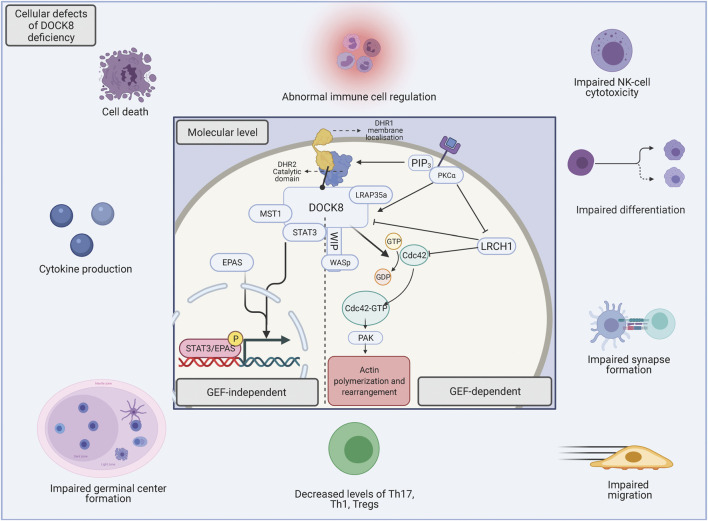
The underlying molecular foundation for DOCK8 deficiency. The perturbation of DOCK8 expression disturbs a broad spectrum of immune cell functions such as differentiation, survival, migration, activation, immunotolerance and -function (McGhee and Chatila, 2010). The basis of the various functions of DOCK8 can be divided into either GEF-dependent actin regulation or functions within GEF-independent pathways. When chemokines bind to extracellular receptors, phosphoinositide 3 kinase (PI3K) is activated and initiates the production of phosphatidylinositol (3,4,5)-triphosphate (PIP3), which recruits DOCK8 via the DHR-1 subunit, which consequently leads to membrane-adjacent GEF activity (Xu et al., 2017; Sakurai et al., 2021). In addition, when chemokines bind extracellular receptors, PKCα is activated, which phosphorylate DOCK8 for dissociation from Leuchine Rich Repeats And Calponin Homology Domain Containing (LRCH1) (2), thus diminishing its inhibitory impact (Xu et al., 2017). The catalytic DHR2 domain of DOCK8 interacts with the nucleotide-free form of the Rho GTPase Cdc42, and mediates activation through GDP-GTP exchange (Harada et al., 2012) and leads to down-stream regulation of several biological activities such as cell morphology, -survival, -signaling and -cytoskeletal dynamics, all mediated through p21-activated kinases (PAK) (Bokoch, 2003). In addition to the aforementioned functions, DOCK8 loss in the GEF-independent pathways leads to nuclear translocation of EPAS1 promoting IL-31 production (Yamamura et al., 2017). Furthermore, DOCK8 associates with the transcription factor STAT3 and facilitates activation-induced STAT3 translocation to the nucleus. Here, the guanine nucleotide exchange function of DOCK8 is also necessary for optimal STAT3 phosphorylation and Th17 differentiation (Su et al., 2019).

The DHR2 domain of DOCK8 acts as a Cdc42-specific GEF (Kunimura et al., 2020) and therefore regulates Cdc42-specific activities such as cytoskeleton remodelling and actin polymerization, which in turn influence diverse signalling pathways and controls cellular morphology, migration, and protein trafficking (Begum et al., 2004; Li and Gundersen, 2008; Kumari et al., 2014) (Figure 2). This has particularly been reported in T and B cells where Cdc42 have been shown to be implicated in cytoskeletal remodelling necessary for functional T cell activation and cytokine secretion as well as defects in B cell receptor signalling and differentiation into plasma cells (Chemin et al., 2012; Burbage et al., 2015; Su and Orange, 2020) Mutations in Cdc42 are associated with an unusual broad spectrum of diverse abnormal phenotypical characteristics which alters morphological appearance and somatic/non-somatic functions. In some patients, cases of immunodeficiency have been reported although the phenotypic spectrum associated with Cdc42 mutations seems wider than that of DOCK8 deficiency (Al-Herz et al., 2016). This would be explained by the ubiquitous expression of Cdc42 whereas DOCK8 expression is largely confined to cells of the immune system, leading to the immune-specific phenotypical characteristics of DOCK8 deficiency^1^.

DOCK8 specifically associates with the transcription factor STAT3, which is mutated in AD-HIES. This interaction facilitates activation-induced STAT3 translocation to the nucleus, and the guanine nucleotide exchange function of DOCK8 is also necessary for optimal STAT3 phosphorylation and Th17 differentiation (Su et al., 2019). This functional relationship between DOCK8 and STAT3 explains the phenotypic overlap between DOCK8 deficiency and AD-HIES.

### Immunological Impairment

There is a large diversity in immunophenotypical appearance of DOCK8 deficiency patients, which reflects the prominent role of DOCK8 in several key immunological processes (McGhee and Chatila, 2010) either in a cytoskeleton-dependent or -independent immune response in both innate and adaptive immunity (Figure 2). DOCK8 therefore serves critical roles in several immune cell types to preserve a broad immune response against bacterial, viral, and fungal agents as well as to sustain self-tolerance.

DOCK8 regulates actin cytoskeletal rearrangement (Dustin, 2002), which has been deemed crucial for facilitating adhesion and formation and functionality of the immunological synapses. This interaction between an immune cell and an antigen presenting cell is mediated by surface components such as the lymphocyte function associated-1 (LFA-1) and the counter receptor Intercellular Adhesion Molecule (ICAM-1), which plays an essential role in the complex cascade of molecular events inducing optimal function and homeostasis of immune cells (Janssen et al., 2016). In the absence of DOCK8, a significant impairment of LFA-1/ICAM-1 binding capacity is observed in CD8^+^ T cells, Regulatory T cells (Tregs), B cells, T follicular helper cells (Tfh), and T helper (Th) cells explaining some of the broad implications of DOCK8 deficiciency (Randall et al., 2011; Randall et al., 2012; Zhang et al., 2016; Janssen et al., 2017; Janssen et al., 2020).

DOCK8 deficiency causes reduced humoral immunity and self-tolerance. The germinal centers, located in secondary lymphoid organs, facilitate the selection and maturation of antigen-activated B-cell clones and provide an optimal immunological response to infections or immunization (Meshaal et al., 2018). However, in absence of DOCK8, the migration of Tfh cells into the germinal center is impaired (Zhang et al., 2016). This may play a significant part in the impaired maturation of B cells into memory cells (Randall et al., 2009; Caracciolo et al., 2014) and reduced persistence of the germinal centers (Biram et al., 2019). Furthermore, the compromised immunological synapse formation (Zhang et al., 2016) and deficient LFA-1 polarization consequently results in reduced production of high affinity IgG antibodies (Jabara et al., 2012; Zhang et al., 2016; Tang et al., 2019), reduced receptor repertoire, and antibody avidity (Janssen et al., 2014).

A heightened immune response, caused by abnormal regulation of T helper and Tregs and increased IgE production, consequently leading to atopic diseases, is common in DOCK8 deficient patients (Aydin et al., 2015). This may partly be due to the significant numerical reduction of Tregs (Caracciolo et al., 2014; Du et al., 2021). Tregs, a subset of CD4^+^ T cells, provide an essential negative immunomodulatory function in immune homeostasis and maintaining immune tolerance towards self-antigens (Singh et al., 2017). In the absence of DOCK8, the capacity of Tregs to suppress the proliferation of T cells is absent causing autoimmunity (Shi et al., 2018; Du et al., 2021). This may be attributed to an impaired function of the role of DOCK8 in IL-2 signalling (Randall et al., 2011; Shi et al., 2018), impaired Treg migration (Randall et al., 2021), and defective thymocyte differentiation to Treg (Janssen et al., 2021). Susceptibility towards atopic diseases may in addition be caused by the bias towards Th2 (Engelhardt et al., 2015), lack of peripheral B cell tolerance, and increases in autoantibody production (Du et al., 2021).

One of the key features of DOCK8 deficiency patients is their predisposition for cutaneous infections (Zhang et al., 2014; Aydin et al., 2015). This may stem from abnormal trafficking of immune cells to the skin as DOCK8-mutated T-cells and NK-cells show impaired morphological integrity leading to cytothripsis during prolonged migration through confined spaces (Lambe et al., 2011). The increased susceptibility to non-skin centred viruses may reflect the progressive lymphopenia, particular of the T cell population, and atypical functionality of T cells due to impaired persistence and recall of antigen-stimulated CD8 T cells, irregular synapse formation with the antigen presenting cells, altered differentiation and impaired proliferation of T cells and DCs (Zhang et al., 2010; Keles et al., 2014; Janssen et al., 2020). In addition, the decreased circulating plasmacytoid dendritic cells and impaired migration of dendritic inhibits normal trafficking to lymph nodes leading to insufficient dendritic cell accumulation in parenchyma for optimal conditions for T-cell priming (Mizesko et al., 2013; Krishnaswamy et al., 2015; Krishnaswamy et al., 2017; Kunimura et al., 2020).

The Natural Killer (NK) cell population exerts an essential antiviral effect by enforcing cellular death of virus-infected cells and is essential for tumour surveillance. The cytotoxic effector function of NK cells in DOCK8 deficient patients is also defective due to impaired lytic synapse formation, abnormal actin accumulation and granule polymerisation (Crawford et al., 2013). Furthermore, DOCK8 is involved in the development of Natural Killer T (NKT) cells and their cytokine production meaning that DOCK8 deficient patients have immature NKT cells and/or NKT cells that display compromised survival (Tangye et al., 2017).

The quantity and function of Th17 T cells is also diminished in DOCK8 deficiency patients (Milner et al., 2008; Caracciolo et al., 2014; Su et al., 2019). Decreased differentiation of Th17 T cells has been accentuated as one of the primary characteristics of HIES (Sandquist and Kolls, 2018). Reduced Th17 differentiation leads to suboptimal activity of anti-fungal and anti-bacterial immunity mainly due to impaired recruitment of neutrophils (Keles et al., 2016). This is partially due to memory CD4^+^ T cells favouring the production of Th2 cytokines at the expense of Th1 and Th17 promoting cytokines (Milner et al., 2008). In addition, intrinsic factors inhibiting Th17 differentiation due to impaired STAT3 phosphorylation, translocation, and transcriptional activity is also implicated (Al-Herz et al., 2016).

## Clinical Manifestations of DOCK8 Deficiency

**FIGURE 3:**
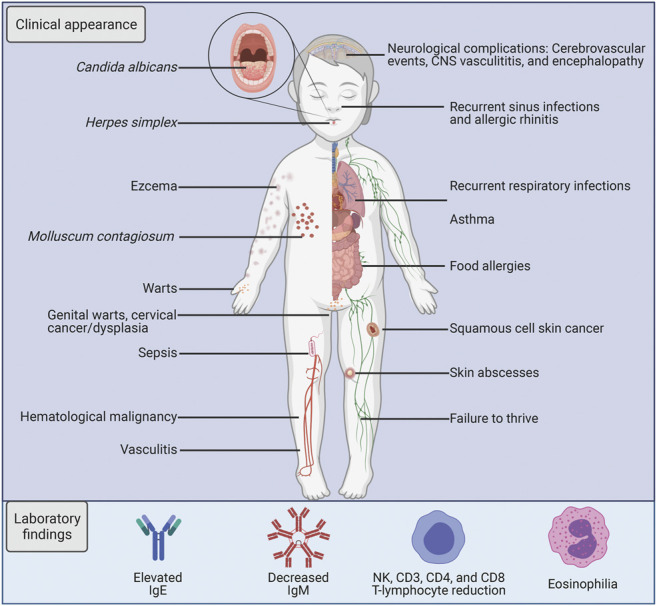
Characteristics of DOCK8 deficiency. A schematic illustration listing the key clinical and laboratory findings in DOCK8 deficiency patients.

The initial diagnosis of DOCK8 deficiency is based on the clinical characteristics in combination with the laboratory immunological findings, with final verification through genetic analysis. DOCK8 deficiency was described clinically for the first time in 2009 and is characterized by early-onset and severe morbidity. Cohort studies have reported around 50% probability to survive beyond 20 years of age with a mean age at death of 9–12 years (Aydin et al., 2015; Zhang et al., 2014). The disease primarily presents with atopic disease, upper and/or lower respiratory infection, frequent viral cutaneous infections, and malignancy (Figure 3) (Zhang et al., 2009; Zhang et al., 2014; Aydin et al., 2015; Haskologlu et al., 2020). Mortality occurs primarily due to infectious agents particularly affecting the skin and respiratory tracts, followed by malignancy, and less commonly CNS vasculitis (Aydin et al., 2015). Almost obligatory findings in these patients are eczema and markedly elevated IgE levels, and there is a high frequency of atopic diseases like food allergies and asthma (Chu et al., 2012; Broides et al., 2017).

Flow cytometric analysis is used to analyse intracellular expression levels of DOCK8, but it is also used to identify B cell maturation arrest and altered frequencies of CD4^+^ and CD8^+^ T cells (Caracciolo et al., 2014). The circulating peripheral blood of DOCK8-deficient patients is characterised by a decreased CD4^+^ T cell count and a shift in the CD8^+^ T cell compartment towards a more exhausted phenotypic subset (Janssen et al., 2020). The B cell compartment displays an increase in naive B cells and a reduction in memory B cells (Tang et al., 2019). However, differential counts of lymphoid cells can show a significant heterogeneity in abnormal findings, thus warranting additional clinical parameters for diagnosis (Alsum et al., 2013).

DOCK8-deficiency leads to a predisposition of cancer, particularly subtypes of haematological or epithelial origin which are often virally-induced either by Epstein Barr Virus (EBV)-driven leiomyosarcomas and lymphomas, and/or Human Papilloma Virus (HPV)-associated squamous cell carcinomas (Papan et al., 2014; Zhang et al., 2014; Aydin et al., 2015; Haskologlu et al., 2020) In the aforementioned cohort studies, 8–17% of patients had developed malignancies during the follow-up period, which included cases of lymphoma (Burkitt and non-Hodgkin lymphoma), squamous cell carcinoma, and sarcoma (Zhang et al., 2014; Aydin et al., 2015).

To summarize, physicians are encouraged to be vigilant about the clinical manifestations of DOCK8-related primary immunodeficiency. It is mainly characterized as HIES and enhances the susceptibility of recurrent viral and bacterial infection, atopic disease, and higher probability of malignancy.

## Current Treatment Strategies

Present management of DOCK8 deficiency includes frequent screening for disease progression and treatment of complications through administration of immunoprophylaxis, antiviral and antibacterial treatments prior to a definitive cure through HSCT, if a compatible donor can be identified.

The evidence supporting allogeneic HSCT for treatment of DOCK8 has been described through multiple reports (Chu et al., 2012; Aydin et al., 2019). HSCT is performed after an initial myeloablative or reduced intensity conditioning regimen consisting primarily of chemotherapy and occasionally with additional radiotherapy (Aydin et al., 2019). The purpose of conditioning is to induce adequate immunosuppression and ablation of the recipient’s hematopoietic stem cells. Administration of interferon alpha has shown efficacious as a rescue therapy for viral infections such as Herpex Simplex virus (HSV) and Human Papilloma virus (HPV) (Al-Zahrani et al., 2014; Gernez et al., 2018). In addition, patients awaiting stem cell transplantation benefit from immunoglobulin replacement therapy (IVIG) and prophylactic treatment (Bazinet and Popradi, 2019). Antibodies in DOCK8 patients have been shown to display reduced avidity, which is why IVIG is recommended despite normo- or hyperphysiological antibody levels (Janssen et al., 2014). Furthermore, prophylactic treatment is given post-transplantation to prevent infection and non-infectious complications in the period until immune reconstitution (Devetten and Armitage, 2007).

The detection and aggressive treatment of infectious disease is paramount to avert fatal progression leading to death. However, a large retrospective report consisting of 136 patients with DOCK8 immunodeficiency accentuates the severe disease progression. Unfortunately, even with early intervention with aggressive therapies or prophylaxis such as anti-bacterial, fungal, viral, immunomodulatory, and immunoglobulin replacement treatment, 63% of the patients succumb by their fourth decade of life (Aydin et al., 2015). Therefore, early allogeneic HSCT is clearly indicated, which is also the sole possibility for a curative treatment. Advancements in HSCT have vastly increased the post-HSCT survival for DOCK8 deficiency patients. Hence, patients undergoing HSCT between 1995 and 2010 had a 2-years overall survival of 57 *versus* 92% for patients transplanted between 2011 and 2015 (Aydin et al., 2019). However, HSCT still entails several risks of severe adverse events, particularly from haploidentical relatives or unrelated donors which pose risks of graft-versus host disease and graft failure (Fox et al., 2018). Reports show that among DOCK8 deficient patients undergoing HSCT, 33% develop acute graft versus host disease (Aydin et al., 2019). Furthermore, HSCT is not always available due to disease progression because of late or misguided diagnosis, and the lack of HLA-compatible donors (Broder et al., 2017; Slatter and Gennery, 2018; Gavrilova, 2019).

Finally, HSCT is associated with high expenses and its use is gradually increasing thus indicating the need for novel therapies that reduce the overall medical cost associated with HSCT and its side effects (Morgan et al., 2017; Mayerhoff et al., 2019). Autologous HSCT with genetically modified stem cells may constitute a promising therapy with fewer adverse outcomes and higher availability to patients with DOCK8 deficiency (Kim et al., 1996; Talib and Shepard, 2020).

## The Therapeutic Promises of Genome Editing

**FIGURE 4:**
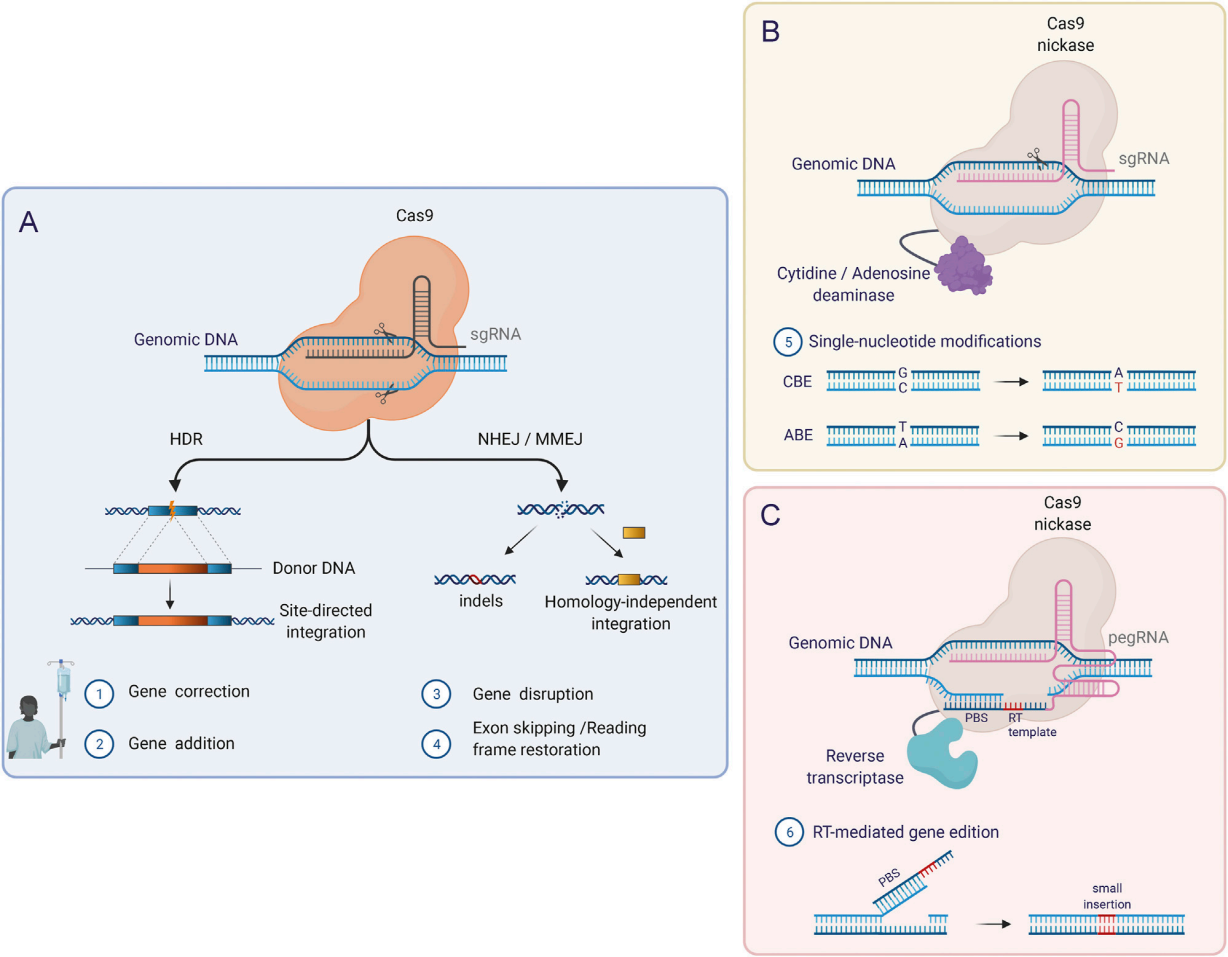
Genome editing tools based on CRISPR/Cas. **(A)** The original CRISPR/Cas RNA-guided nuclease system induces DSBs in the genome in a targeted manner and requires binding of the single guide RNA molecule (sgRNA) to the target DNA as well as the recognition of a specific PAM sequence adjacent to the target sequence. Using this system, the two major DNA repair pathways (HDR and NHEJ) can be exploited to introduce modifications at the target locus. **(B)** Base Editors (BEs) combine Cas nickases lacking one nuclease domain with DNA deaminases. BEs mediate single-nucleotide conversion, which enables the correction of point mutations. Two types of BEs can be distinguished: Cytosine base editors (CBEs), which mediate C-G to T-A conversions, and adenine base editors (ABEs) which induce A-T to G-C conversions. **(C)** Prime Editors (PEs) can induce point mutations, small insertions, and small deletions and consist of a Cas nickase fused to a reverse transcriptase (RT) domain. In this system, a reformulated prime editing gRNA (pegRNA) confers the specificity (like the sgRNAs) but additionally contains the template for the desired DNA modifications in the 3′ end. After the induction of a single-strand break (SSB) by the Cas9 nickase, the 3′ end of the pegRNA (primer binding site; PBS) hybridizes with the free 3′ DNA end and acts as a reverse transcription template.

Our ability to precisely rewrite and manipulate the instructions encoded in the genome has greatly expanded over the last few decades. By using programmable nucleases, such as Zinc Finger Nucleases (ZFNs) (Miller et al., 2007; Szczepek et al., 2007; Christian et al., 2010), TALE nucleases (TALENs) (Miller et al., 2011; Mussolino et al., 2011; Jinek et al., 2012) and RNA-guided Cas nucleases (Cong et al., 2013; Tebas et al., 2014), researchers can direct the creation of double-strand breaks (DSBs) to specific sites in the DNA and hereby exploit the cellular DNA repair machinery to introduce desired genetic modifications. Induced DSBs are repaired through either Non-Homologous End-joining (NHEJ), an error-prone repair mechanism that induces insertion or deletions (INDELs) at the DNA breakpoint, or Homology-Directed Repair (HDR), a precise repair pathway that uses homologous repair templates to copy from during repair of the DSB, allowing the inclusion of foreign DNA sequences into a specific locus (Figure 4A).

Using programmable nucleases, gene disruption or remodeling of regulatory sequences can be easily achieved by NHEJ. These are strategies that have already been used in clinical trials with promising results (Bushman, 2007; Xu et al., 2019; Stadtmauer et al., 2020; Frangoul et al., 2021; Gillmore et al., 2021). However, for most autosomal recessive PIDs, the addition, replacement, or correction of the affected gene is required thereby necessitating the use of the HDR pathway. This can be achieved by direct correction of the genetic mutation or through the integration of the complete or partial open reading frame cDNA sequence either directly after the endogenous promoter or into a safe harbor site in the genome with a heterologous promoter.

The ability of hematopoietic stem cells (HSCs) to perpetually self-renew and differentiate into all hematopoietic lineages makes them an ideal therapeutic target for gene therapy for treating blood and immune system diseases, including PIDs. Several gene therapy clinical trials have been performed in HSCs since the first one in 1990, and they have mainly been performed using retroviral vectors that integrate the transgene into the chromosomes of HSCs in a semi-random fashion (Booth et al., 2016). Despite clinical trials for several PIDs showing curative potential of these gene therapies, there are also multiple reports of patients developing leukemia due to insertional mutagenesis caused by the retroviral vector (Dever et al., 2016) These events were caused by vector integration close to cancer-related genes such as LMO2 and transactivation of these genes by the strong viral promoter/enhancer elements present in the retroviral vectors. Although lentiviral vectors with self-inactivating mechanisms have proven a safer alternative, they have not entirely eliminated the risks associated with random vector integration, thereby rationalizing the pursuit for safer alternatives such as precise genome editing. Undoubtedly, the advent of the CRISPR/Cas gene editing system supports the onward march towards precision gene therapy.

Initially the CRISPR/Cas system was comprised of the Cas9 endonuclease and two small RNAs: CRISPR RNA (crRNA) and *trans*-activating RNA (tracrRNA). While crRNA confers specificity to a complementary region in the genome and thereby serves to guide Cas9 to its target, the tracrRNA acts as a Cas9 binding handle to enable the formation of a ribonucleoprotein (RNP) complex. In seminal work from the 2020 Nobel prize winners Jennifer Doudna and Emmanuelle Charpentier they merged the crRNA and tracrRNA into a single guide molecule (sgRNA) thereby reducing the system from three to two components (Cong et al., 2013).

The therapeutic potential of targeted gene editing in long-term repopulating HSCs (LT-HSCs) was first demonstrated in a preclinical study for X-linked Severe Combined Immunodeficiency (SCID-X1) using ZFNs delivered by mRNA electroporation and repair template delivery by an integration-defective lentiviral vector (IDLVs). In this study, Genovese et al. achieved targeted integration of a partial IL2RG cDNA comprising a super-exon of exons 5–8 of IL2RG into exon 5 of the endogenous IL2RG gene. Thus, transcription occurs from the endogenous IL2RG promoter and exon 4 splices with the newly inserted super-exon to generate a correct and full-lenght IL2RG reading frame, thereby providing a platform with the potential to correct all SCID-X1 IL2RG mutations downstream of exon 4. Long-term engraftment of the targeted HSCs in transplanted NSG mice was confirmed and they were also able to correct the defective IL2RG gene in HSCs from a patient with SCID-X1. The CRISPR/Cas system was similarly applied in HSCs for the first time to correct the mutation in the β-globin gene responsible for Sickle Cell Disease (SCD) (Lattanzi et al., 2021). Here, precise correction of the disease-causing mutation was performed with similar evidence of long-term engraftment in mice and reconstitution of functional β-globin (Mohrin et al., 2010). However, both studies also revealed what is now considered the largest challenge for applying precise HDR-mediated gene editing in HSCs, which is the low efficiencies of HDR-mediated editing in the long-term (LT)-HSC compartment compared to the progenitor cell population of the total CD34^+^ cells. This is mainly believed to be caused by HDR only being active in the late S and G2 phases of the cell cycle whereas NHEJ is the prevailing repair mechanism in quiescent cells (Wilson et al., 2008). This poses a paradoxical challenge in HSC-based gene editing since otherwise quiescent HSCs must be forced into cycling, but cycling is known to be associated with loss of stem cell properties (Song et al., 2016). Hence, NHEJ-focused HSC therapies have shown higher efficiencies in HSCs, confirmed in a recent clinical trial (Frangoul et al., 2021), but is generally more difficult to apply for recessive disorders where expression of a functional gene must be restored.

To enhance HDR frequencies, researchers have been working on different strategies that include the use of repair pathway-modulating small molecules that promote HDR or inhibit NHEJ (Lin et al., 2014; Chu et al., 2015; Robert et al., 2015; Nambiar et al., 2019; De Ravin et al., 2021; Fu et al., 2021), cell cycle synchronization to ensure S/G2 status upon editing (Charpentier et al., 2018; Shin et al., 2020), and the development of novel engineered Cas9 variants for example fusing HDR-promoting or NHEJ-inhibiting proteins to Cas9 (Jayavaradhan et al., 2019; Ferrari et al., 2020). New protocols also transiently inhibit the p53 pathway to achieve high percentages of HDR editing in LT-HSCs (Vavassori et al., 2021). The most advanced example of this is a recent study correcting the CD40 ligand gene (CD40LG), which has deactivating mutations in X‐linked hyper‐IgM syndrome type I (HIGM1) (Schiroli et al., 2019). Here, the authors introduce mRNA encoding a dominant negative p53 variant (GSE53) along the CRISPR/Cas9 gene editing components. Prior studies have shown that during gene editing in HSCs, DSBs and the presence of adeno-associated virus (AAV) vector genomes, used to deliver the HDR repair template, activate p53, which constrains HSC yield, proliferation, and engraftment of gene-edited HSCs (Cromer et al., 2021). With the addition of GSE53, the authors showed up to 30% CD40LG correction frequencies (cDNA integration) in LT-HSCs.

The HDR pathway also provides the possibility of replacing entire gene sequences or large genomic regions. This was recently showcased in LT-HSCs where a DNA repair template was designed in a way that the copy-paste mechanism replaced the one pf the α-globin genes (HBA1) with that of β-globin. This approach could prove therapeutic in patients with β-thalassemia to normalize the balance between α and β chains, thus restoring adult hemoglobin functionality (Maresca et al., 2013). Moreover, this study showed that whole gene replacement is possible in LT-HSCs, thereby providing an additional genome editing strategy for genetic diseases.

As alternatives to HDR, novel gene correction approaches based on HDR-independent targeted gene integration (Sakuma et al., 2016; Yao et al., 2017; Porto et al., 2020), base editing (Anzalone et al., 2019), and more recently prime editing (Newby et al., 2021) try to overcome the inherent limitations of HDR-mediated genome editing. In base and prime editing, the functional properties of Cas9 can be extended by fusing new effector domains to catalytically inactive Cas9 protein or Cas9 nickase. In this way, without requiring DSBs or donor DNA templates, Base Editors (BEs) mediate single-nucleotide conversions in a targeted manner, while Prime Editors (PEs) write new genetic information into a specific nicked locus directed by a small template present on the sgRNA (Figures 4B,C) (Newby et al., 2021). These are promising alternatives for genome editing, and BEs have already shown as high as 68% base editing of the β-globin gene in human LT-HSCs evaluated 16 weeks after transplantation into mice (Vaidyanathan et al., 2018) Similar evidence for PEs must be provided to reinforce their applicability in HSCs.

### CRISPR/Cas9 Delivery Strategies in HSCs

Delivery has for a long time been the main hurdle for advancing gene therapy. Since HSCs were the first stem cells to be discovered, purified, and used for therapy (bone marrow transplants), HSCs were also obvious first choice for gene therapy since *ex vivo* gene therapy is much simpler than *in vivo* gene therapy.

In general, three different approaches exist to introduce the two components of the CRISPR/Cas-system into cells. In the first approach, DNA such as plasmid DNA is used, encoding the Cas9 and sgRNA. Plasmid delivery is associated with rather slow onset of editing, when compared to the other modalities (Vaidyanathan et al., 2018). The second “all-RNA” approach delivers mRNA encoding Cas9 along in vitro-transcribed or chemically synthesized sgRNAs (Laustsen et al., 2019). Lastly, a recombinant Cas9 protein precomplexed to the sgRNA as an RNP complex can be delivered into cells (Genovese et al., 2014; Lino et al., 2018; Vakulskas et al., 2018). However, in most primary cells, the cost-effective plasmid delivery approach leads to high undesirable cytotoxicity (Cromer et al., 2018; Lino et al., 2018). All-RNA delivery using Cas9 mRNA and sgRNAs is better tolerated by primary cells, even though they still induce a higher innate immune response than RNP delivery (Hendel et al., 2015). Hence, the preferred delivery format for HSC gene editing is using RNP complexes with sgRNAs that are chemically synthetized with modified nucleotides at both ends to protects them from degradation by exonucleases (Laustsen et al., 2019). The delivery mode of choice in HSCs is using electroporation, which relies on short pulses of electrical current to induce small pores in the cell membrane that allows diffusion of macromolecules (Gundry et al., 2017). Combined with Cas9 RNP, this mode has shown exceptionally high on-target efficiency. At the same time, the short half-life of the RNP complex provides a hit-and-run modality that reduces the risk of off-target activity at sites that resemble the intended target in the genome (Genovese et al., 2014; De Ravin et al., 2017; Lino et al., 2018; Vaidyanathan et al., 2018).

In addition to the Cas9 and sgRNA, HDR requires the introduction of a repair template. Here, non-viral repair templates like chemically synthesized single-stranded oligodeoxynucleotide (ssODN) of up to 200 nt have shown effective in HSC gene editing (DeWitt et al., 2016; Romero et al., 2019). However, ssODNs have been reported to be less efficient and induce higher toxicity compared to repair template delivery approaches that rely on viral vectors. At the same time, ssODNs suffer from size constraints associated with chemical DNA synthesis (Roth et al., 2018). However, recent work establishes evidence for surpassing the size constrains of ssODNs enabling delivery of >1 kb long dsDNA co-electroporated with Cas9 RNP complexes in primary human T cells with a tolerable toxicity profile (Wang et al., 2015). The applicability of this platform in HSC is intriguing, but needs further investigation. Lentiviral vectors, which have been employed in numerous clinical trials for *ex vivo* HSC gene therapy, have also been employed as repair template for HDR. Here, the natural integrating mechanism of lentiviral vectors is removed by introducing an inactivating mutation in the viral Integrase enzyme to generate integration-defective lentiviral vectors (IDLVs) suitable for donor DNA delivery. IDLVs do have higher carrying capacity than AAV vectors, but have been shown to be inferior to AAVs when used for repair template delivery, where specifically AAV serotype 6 has proven effective in HSCs with tolerable cytotoxicy (Grieger and Samulski, 2005). Despite the relatively low packaging capacity of AAV vectors at around 4.5 kb (Bak and Porteus, 2017), this is sufficient for most genome editing purposes, and an approach splitting a large transgene between two AAV donors that undergo sequential HDR at the target locus has been devised to overcome this limit (Bak et al., 2018b). Hence, the combination of RNP complexes with simultaneous delivery of DNA donor template using AAV6 currently represents the most promising technology for versatile gene editing in HSCs (Hacein-Bey-Abina et al., 2002; Bak et al., 2017; Wilkinson et al., 2021).

## Towards a Curative CRISPR/Cas9-Based Gene Editing Approach for DOCK8-Related Primary Immunodeficiency

Retroviral gene therapy in autologous HSCs has provided clinical benefit in several PIDs including SCID-X1 (Aiuti et al., 2002; Gaspar et al., 2004; Hacein-Bey-Abina et al., 2010; Gaspar et al., 2011), Adenosine deaminase deficiency (ADA-SCID) (Aiuti et al., 2009; Boztug et al., 2010; Cicalese et al., 2016; Scott et al., 2017), Wiskott Aldrich Syndrome (WAS) (Ott et al., 2006; Aiuti et al., 2013; Hacein-Bey Abina et al., 2015), and X-linked chronic granulomatous disease (X-CGD) (Cavazzana-Calvo et al., 2010; Kang et al., 2010). However, integration of the transgene in these approaches occurs semi-randomly into the patient’s genome, and as mentioned earlier this can lead to oncogenic transformation due to insertional mutagenesis. Even vectors with lower genotoxic potential, such as lentiviral vectors (LVs), can give rise to insertional mutagenesis, which is a risk that needs to be considered in clinical applications (Brown et al., 1998; Modlich et al., 2009). Furthermore, transgene expression levels often differ from the physiological levels of the affected gene due to the use of a constitutive promoter that does not allow tissue-specific or temporal regulation of expression. Unregulated gene expression can for some diseases be detrimental exemplified by the CD40LG gene, which must be expressed at controlled levels as evidenced by preclinical mouse studies where *ex vivo* gene therapy in a mouse model of X-linked hyper-IgM syndrome with CD40LG-encoding murine gamma-retroviral vectors in HSCs led to lymphoproliferative disorder assumingly as a consequence of unregulated expression of the CD40L transgene (Glessner et al., 2017). DOCK8 deficiency has not been approached with retro- or lentiviral gene delivery, but copy number variation analyses have identified DOCK8 duplications to be significantly associated with a spectrum of neuropsychiatric disorders (Jing et al., 2014). Even though a direct link from DOCK8 CNV to immunological defects has not been established, this might suggest that elevated levels of DOCK8 gene expression can impede normal cellular function. Tight DOCK8 expression in different mature immune cell subsets and regulated DOCK8 expression during hematopoiesis would be impossible to establish with LVs carrying constitutive heterologous promoters and would require full delineation of the regulatory mechanisms that govern DOCK8 expression and reconstruction of a DOCK8 promoter suitable for LV use. Hence, LV-mediated gene delivery may not be a therapeutic option in DOCK8 deficiency, whereas precise gene editing approaches may be optimally suited for such diseases. In the following section, we summarize essential considerations for gene editing and describe different gene editing strategies and their potential use for correcting DOCK8-mediated immunodeficiency.

### Potential Gene Correction Strategies for DOCK8 Deficiency

#### Utilizing the Non-Homologous End-Joining Pathway

An NHEJ-based treatment strategy for DOCK8 deficiency would be highly desirable since the NHEJ pathway is much more active in HSCs compared to the HDR pathway. However, due to the autosomal recessive nature of DOCK8 deficiency, an NHEJ-based strategy that introduces INDELs in the genome is challenging to apply to DOCK8. For disease-causing variants where the reading frame is disrupted, one option for the NHEJ pathway is to use the “reframing” approach which relies on introduced INDELs to restore the correct reading frame (Figure 4A). However, CRISPR/Cas-generated INDELs occur in a semi-stochastic fashion, which means that a population of edited cells will contain a mix of different INDELs, which are specific to the sgRNA used. Hence, this approach depends on the availability of a sgRNA in the vicinity of the mutation, which creates reframing INDELs. Depending on the type and location of these reframing INDELs there may be a loss or addition of amino acids to the reading frame, which may in some rare instances establish a dominant gain of function variant. However, the frequency of reframing INDELs may only need to be low, as evidenced by the aforementioned cases of somatic reversion establishing the possibility that correction of a single lymphoid progenitor or stem cell may be sufficient to provide therapeutic benefit. Depending on the type and location of these reframing INDELs there may be a loss or addition of amino acids to the reading frame, which may perturb protein function. However, for some patient mutations, this approach may be applied. Recent preclinical studies have in fact used reframing to correct mutations in HSCs from patients with Fanconi Anemia where there is a great survival adantage (Román-Rodríguez et al., 2019). The downside of this approach is that it cannot be generalized but would need a different sgRNA for each patient mutation. Also, for longer gene deletions, this approach would not be feasible and 61.5% of patients harbour deletions. This individualized approach is costly to develop and therefore difficult to bring to clinical trials.

#### Base and Prime Editing

BEs are promising new tools for gene editing, but they can only address a subset of mutations (Figure 4B). In theory, Cytosine BEs enable correction of 26% of all known pathogenic SNP variants, while the Adenine BEs could potentially correct 28%. However, in DOCK8 deficient patients, only a subset of approximately 26% of patients carry pathogenic SNPs in DOCK8. Therefore, even the pursuit to develop individualized base editors for specific patient mutations would only be possible for a subset of the patients. Similar challenges exist for the recently developed PE (Newby et al., 2021) (Figure 4C). The most distinctive attribute of this technology and advantage over BEs is its ability to make any small sequence changes. Like the BEs, this occurs without inducing a DSB - thus mitigating the error-prone NHEJ pathway and the low rates of HDR in post-miotic cells (Scholefield and Harrison, 2021). However, PE is limited to make insertions of less than 80 bp and deletions smaller than 50 bp (Kim et al., 2014). This only enables correction of around 15% of the current DOCK8 patient mutations. Furthermore, there have not been any reports of efficient PE in HSCs.

#### Homology-Independent Targeted Insertion

**FIGURE 5:**
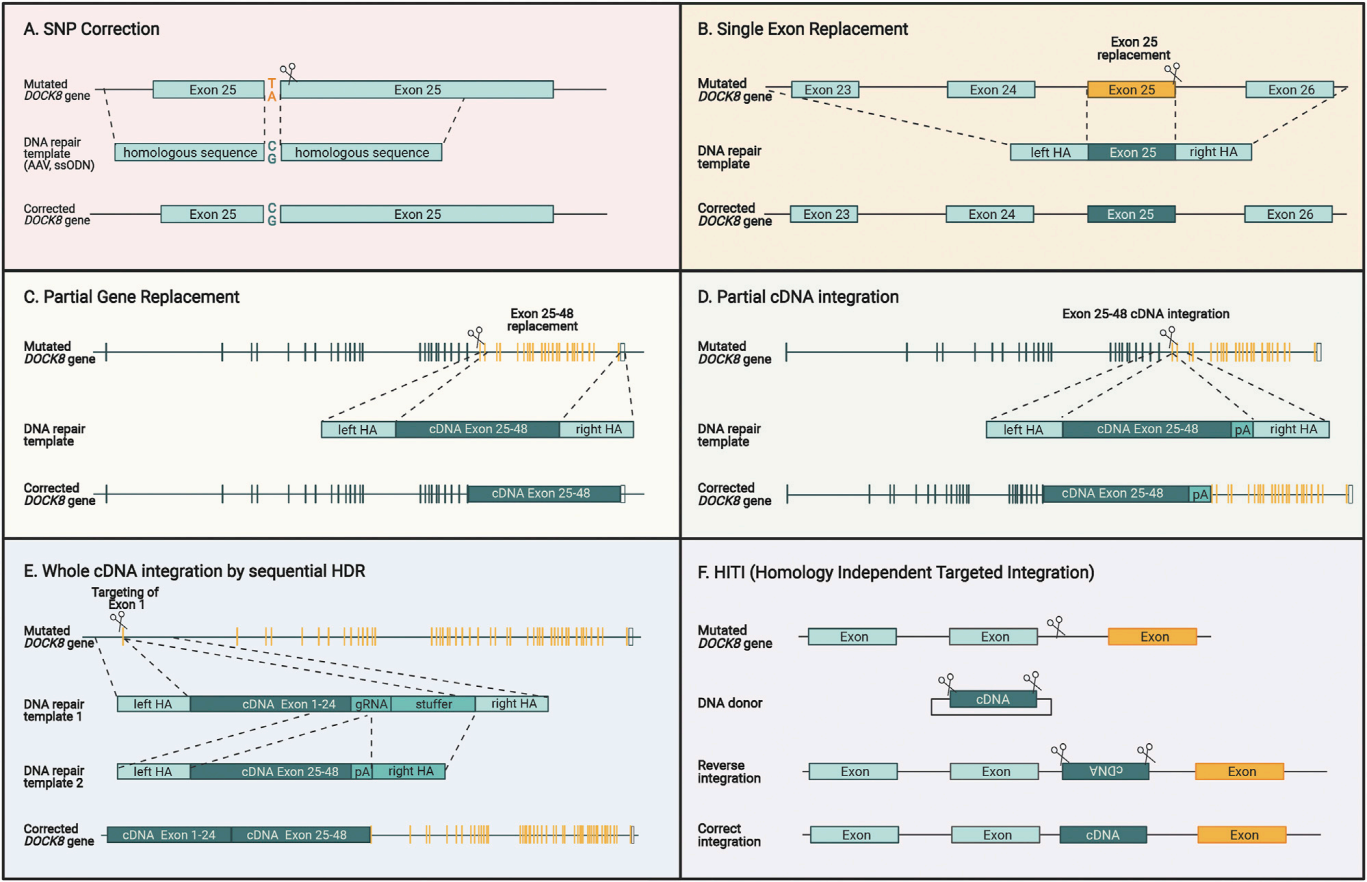
Different genome editing strategies for restoring DOCK8 gene expression. **(A)** Specific correction of SNPs by providing DNA repair templates with homologous sequences surrounding the mutation. **(B)** Single exons can be replaced by introducing a double strand break close to the end of the exon in addition to supplying a DNA repair template encoding homologous sequences surrounding the exon. **(C)** Multiple exons can be replaced by cutting close to the region which is intended to be replaced and providing a DNA repair template with homology arms that are homologous to the adjacent regions. **(D)** A cDNA sequence covering several exons can be introduced upstream to the mutated sequence and the homology arms contain the sequences surrounding the cut site. This way the cDNA will be fused directly to the previous exons, and the downstream exon will be inactivated. **(E)** To replace the entire DOCK reading frame, a two-step HDR approach can be applied due. For utilizing AAV vectors for repair template delivery, this two-step approach is necessary due to the large size of the gene and the limited capacity of AAV vectors. **(F)** HITI is different to the previous HDR-based strategies since it uses the NHEJ pathway to insert a DNA sequence without homology arms directly at the cut site. Since this insertion is not exact, intronic regions are targeted to insert a desired sequence that maintains the normal splicing mechanisms and keeps endogenous expression intact. For all figures, the yellow sequences designate exons where patient mutations will be corrected by the specific approach.

Homology-independent targeted insertion (HITI) is a gene editing approach that enables integration of DNA at a specific target site without relying on homologous sequencies in the DNA donor (Figure 5F). Instead, it uses the NHEJ pathway to integrate the linear DNA donor at the break site through an end-joining mechanism, but its efficiency has been reported to be less than 5% in most cases (Suzuki and Izpisua Belmonte, 2018). The applicability of HITI in CD34^+^ HSPC might be high as these quiescent cells are often in the G0/G1 phases of the cell cycle where NHEJ is the primary DNA repair pathway (Suzuki and Izpisua Belmonte, 2018). Recently, Hanan Bloomer et. al. utilized the HITI system reaching an average of 21% integration in long-term repopulating HSC in mouse xenotransplantation studies (Bloomer et al., 2021). However, the integration mechanism allows transgene cassette insertion in both orientation and the NHEJ mechanism can also lead to end-trimming of the DNA donor template and/or the genomic target site. Hence, this pathway does not lead to exact genome editing outcomes, which might impede its application for DOCK8 deficiency. At present, the technology would need to mature and further studies would need to be conducted to proof its applicability in HSCs.

#### Homology-Directed Repair

The most versatile method in terms of possibilities of gene editing outcome is utilizing the HDR pathway, which requires co-delivery of a homologous DNA repair template. In this template, the new DNA sequence is flanked by DNA sequences that are homologous to the target gene sequence around the cut site (Figure 4A). HDR enables various kinds of genetic alterations ranging from single base pair changes to whole cDNA insertion strategies. In contrast to gene delivery by retroviral vectors, HDR preserves endogenous DOCK8 gene regulatory elements with the possibility to re-establish physiological gene expression levels.

Single base pair correction has previously been shown in CD34^+^ HSCs and is particularly advanced for Sickle Cell Disease (Magis et al., 2019; Lattanzi et al., 2021) This disease is one of the most prevalent genetic disorders and is caused by a single nucleotide substitution that changes a glutamic acid into valine. A direct base pair correction approach is highly desirable since no major perturbations are made to the gene and all regulatory elements of the promoter, untranslated regions, splice elements, and introns are maintained (Figure 5A). This approach could also be applied to DOCK8 mutations but suffers from the same challenge as base and prime editors as mutation-specific CRISPR/Cas reagents must be developed, constituting a major financial burden.

A more universal approach would be to insert part of the DOCK8 reading frame into the endogenous DOCK8 locus thereby spanning larger segments of the gene and potentially covering several patient mutations. This could be used to replace single exons, multiple exons, and potentially all exons (Figures 5B–D). Such strategies have been used preclinically before for multiple hematopoietic diseases including β-thalassemia (Lattanzi et al., 2021), X-SCID (Cromer et al., 2018; Pavel-Dinu et al., 2019), hyper-IgM syndrome (Hubbard et al., 2016; Kuo et al., 2018; Schiroli et al., 2019), and X-CGD (De Ravin et al., 2016; DeWitt et al., 2016). Since AAV is the preferred vector for delivery of the repair template, DOCK8 represents a particular challenge due to its large ORF size of 6.3 kb. The maximum size of AAV packaging is around 4.7kb, which must include the homology arms, which are normally 2 x 400bp leaving around 3.9 kb for the cDNA to be inserted. For DOCK8, this would allow inserting approximately 60% of the DOCK8 ORF. To include as many patient mutations as possible, the optimal region to target would be exons 25–48 encompassing 39% of the known patient mutations (Figure 5D).

We have previously devised an HDR strategy for integrating large gene segments exceeding the capacity of AAV6 vectors. Here, the large transgene is delivered using two separate AAV repair template vectors (Bak et al., 2018b). This makes use of two consecutive HDR steps that first integrate one half of the gene and then the next half, hence overcoming the capacity limit for a single AAV, which potentially would enable a universal HDR approach to target all known DOCK8 mutations present in the reading frame (Figure 5E) (Balakrishnan and Jayandharan, 2014). This approach has recently shown promise as a curative correction strategy for cystic fibrosis, which involves the large 4.4 kb CFTR ORF (Vaidyanathan et al., 2021).

One key aspect is tailoring the donor design and targeting strategies for optimal expression of the target gene. Studies show that for some genes, mere integration of the full reading frame cDNA into the start codon of the endogenous locus leads to suboptimal gene expression levels. Physiological transgene expression can be reached by improving several steps including biallelic integration rates, cDNA codon usage, and inclusion of transcriptional and posttranscriptional regulatory elements. Some studies have used the cDNA of the reading frame only, thereby excluding the 3′UTR from the constructs. This exclusion has advantages during HDR since the 3′UTR sequence in the DNA donor template cannot be diverged from the endogenous 3′UTR like is possible for the reading frame using synonymous codons. This “internal homology” creates two sites of homology between the DNA donor template and the genome: (1) the site of the DSB to which the homology arms have homology and (2) the 3′UTR which is distant from the DSB when designed for the region around the endogenous start codon. This double homology creates the possibility of unpredictable HDR events, which is the reason why the exclusion of the 3′ UTR is preferred. However, 3′ UTRs are known to include several regulatory elements like miRNA binding sites and AU-rich elements that can be stabilizing or destabilising to the mRNA. An example of this is gene editing with CD40LG replacement by HDR where the inclusion of the 3′ UTR is essential to ensure high gene expression levels (Hubbard et al., 2016; Kuo et al., 2018). Studies have also shown that retaining intron one or the terminal intron in the cDNA can contribute positively to efficient expression levels (Gray et al., 2021; Sweeney et al., 2021).

### The Societal Challenges of Bringing CRISPR/Cas9 Gene Therapies for DOCK8 Deficiency to Patients

The European Commission defines rare diseases as those with a prevalence below five of every 2,000 people (<0.25%) (European Commission (2021, 2021). Although no official definition of “ultra-rare” disease has been established, the European Union defines orphan medicinal products as those that address individuals affected by severe or life-threatening diseases which affect no more than 5 persons in 10,000 (<0.05%) withing the European union. Therefore, based on the current literature entailing DOCK8 deficiency diagnoses, this disease may be defined as an ultra-rare disease.

**FIGURE 6:**
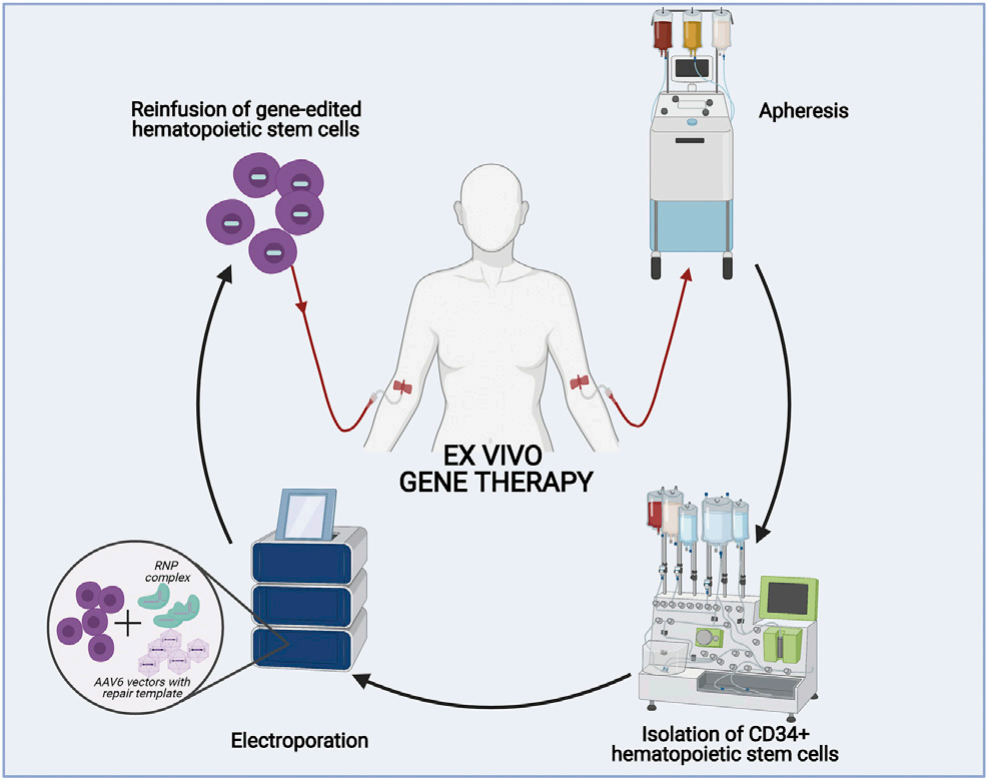
Principles of *ex vivo* gene editing. For a patient specific treatment, CD34^+^ hematopoietic stem cells are isolated from a patient’s blood through apheresis followed by an automated cell processing system that enrich CD34^+^ cells. Afterwards, the genome editing tools (Cas9 and sgRNA) are delivered to the cells by electroporation and the DNA repair templates are delivered by addition of AAV6 vectors to the cell culture. The genetically modified cells can be cryopreserved before infusion into the patient, which has typically undergone myeloablative conditioning prior to infusion.

Several financial, logistical, and ethical questions arise when considering orphan drugs for rare diseases. There are few to no financial incentives for biopharmaceutical companies to venture into ultra-rare disorders due to high development costs and the prospect of low revenues, which is only circumvented with soaring treatment prices. This was demonstrated, with the recently approved one time curative gene replacement therapy for Spinal Muscular Atrophy, which cost more than two million US dollars (Dean et al., 2021). Furthermore, non-economic aspects such as the need for accelerated approval of orphan drugs and how to encourage cooperation between countries and stakeholders in the pursuit of bringing orphan drugs to the marked is worth considering (Kacetl et al., 2020). There has been rapid growth in orphan drug policy establishment, and it is important that governments establish incentives that promote research and development for these indications (Chan et al., 2020). For ultra-rare disease gene therapy development, it is pivotal that translational research and clinical trials are performed in international collaborations to promote access to patient samples and ultimately to centralize clinical trials and coordinate logistical challenges. While this might be possible in high-income countries, several stakeholder are now recognizing that the single largest challenge will be providing access to novel and expensive treatment modalities for patients from low- and middle-income countries and patients from disadvantaged communities and ethnic groups (Gene, 2021). Because gene therapies involve complex procedures during the GMP-compliant manufacturing of a living cell product (Figure 6), there is a need for advanced infrastructure and highly educated personnel. This demand constitutes a major bottleneck for many institutions with the desire to treat patients with novel gene therapies, and they often fall short in meeting the demands from patients who are in critical need to gain access to potential life-saving therapies. In the future, semi-automated closed cell manufacturing systems might make it easier to implement gene therapies locally (Adair et al., 2016).

As the possible applications of gene therapy exceed beyond the field of research, ethical concerns arise and are discussed to establish a framework for appropriate application of genetic therapies. One important ethical consideration in gene therapy is the distinction between somatic and germline gene editing. The 2018 reports of gene edited Chinese twin babies sparked multiple calls for a global moratorium on clinical uses of human germline editing (Lander et al., 2019). Important discussions arose from this event concerning ethical issues such as patient safety, missing consent from the unborn child, uncertainty of the monitoring period of adverse events, how to justify the defying of natural order, and the potential future implementation of gene therapy as a preventive treatment (Araki and Ishii, 2014). While these discussions on germline gene therapy are ongoing, it is important to remember that somatic gene therapy is bringing about an increasing number of success stories, thereby challenging the need for germ line therapies.

### Current Technological Challenges of Gene Editing Reaching the Clinical Setting

Human cells have acquired several mechanisms to detect and correct genomic lesions as each cell experiences several thousand DNA lesions daily (Carusillo and Mussolino, 2020). Most gene editing technologies piggyback on these mechanisms, but also suffer from the adverse events of activating DNA damage responses. This is not only caused by DNA double strand breaks but are also invoked by exposure to AAV vectors. This can lead to cumulative p53 pathway activation which can negatively impact engraftment of edited HSCs (Cromer et al., 2021).

Off-target INDEL induction, translocations, chromothripsis, large on-target INDELs, and off-target integration of DNA donor template are other non-intended consequences that can potentially lead to adverse events and must therefore be evaluated (Urnov, 2021). Off-target activity is based on following three elements: the uniqueness of the target site, the chromatin state of the genome, and nuclease exposure duration and efficiency. Cas9 specificity is dependent on target homology with the 20-nt spacer region of the gRNA, but this sequence can tolerate a mismatch of several bases, consequently enabling possible binding to secondary unintended regions (Mali et al., 2013). In addition, off-target activity is more common to occur at open chromatin regions rather than at closed chromatin region (Singh et al., 2015; Kim and Kim, 2018). Minimizing the off-target activity can theoretically be reached through either increasing the nuclease dissociation from Watson-Crick base paired genomic regions or reducing its cleavage rate (Bisaria et al., 2017). Several Cas9 variants have been engineered with such overall reduction in DNA affinity, thereby maintaining on-target affinity within a window of maximal cleavage while reducing off-target affinity with concomitant reduction in cleavage (Genovese et al., 2014). Several unbiased detection methods have been developed to identify off-target site, like GUIDE-seq and DISCOVER-Seq (Tsai et al., 2015; Wienert et al., 2019) while other methods like CAST-seq evaluates translocations and other gross rearrangements (Turchiano et al., 2021). Overall, with careful sgRNA design and implementation of novel technologies like high-fidelity Cas9 proteins, these adverse genomic events can be reduced and are often benchmarked against existing lentiviral vectors that integrate their cargo semi-randomly in the genome. Off-target activity and insertional mutagenesis remain important concerns in gene therapy and more clinical trials and long-term follow-up is the only means to truly gauge the proportions and clinical relevance of these events.

## Conclusion and Future Direction

In the near future, we expect several genetic diseases such as DOCK8 deficiency, which lack efficient and safe treatment regimens, to be treatable with appropriate gene therapy strategies. The great advance compared to conventional pharmaceutical approaches is that gene therapy directly corrects the underlying genomic abnormality of the disease. This provides the possibility to cure these diseases rather than treat symptoms. Given the vast diversity in gene-editing tools, we emphasize the requirement of selecting proper therapeutic strategies that match the underlying genetics of the disease. These strategies have distinct advantages, limitations, and potential adverse effects and for many indications the choices might not be straight-forward. Therefore, when pursuing a curative treatment for DOCK8 deficiency it is imperative that several modalities are explored at the developmental stage to maximize the therapeutic effects while considering disease mechanism, mutation locations, and strategies for delivery and correction. We suggest that efficient and patient-universal correction may be achieved by exploiting the HDR pathway. Additionally, *ex vivo* introduction of repair templates using AAV donor templates currently seems to be the most proper delivery mechanism and may be designed to replace single or multiple exons with hot-spot mutations or perhaps even include a full cDNA through a two-step HDR strategy. However, further experimental work is warranted to evaluate the efficiency of the different strategies in pursuit of a definitive cure for DOCK8 deficiency. Meanwhile, intensive research in genome editing leads to a continuous emergence of technologies that are bound to enable future breakthroughs in clinical gene therapy. Already, CRISPR/Cas9-based clinical trials have proven successful for sickle cell disease, beta-thalassemia, and transthyretin amyloidosis (Frangoul et al., 2021; Gillmore et al., 2021), and many efforts are focused on optimizing the conditioning regimen, such as the development of anti-CD117 antibodies that deplete HSCs in a targeted and safe manner (Kwon et al., 2019). With these advances and accumulation of experience from clinical trials, the future looks bright for bringing more CRISPR/Cas9-based gene therapies to patients that are safer and more efficient.

## Data Availability

The original contributions presented in the study are included in the article/Supplementary Material, further inquiries can be directed to the corresponding author.

## Footnotes

1
Biogps.org
